# Testicular Torsion in the Undescended Testis of a Four-Year-Old: A Delayed Diagnosis

**DOI:** 10.7759/cureus.51664

**Published:** 2024-01-04

**Authors:** Leena K Alshaibani, Sara K Alshaibani

**Affiliations:** 1 General Practice, King Hamad University Hospital, Seef, BHR; 2 Pediatric Radiology, King Fahad Specialist Hospital, Dammam, SAU

**Keywords:** cryptorchidism, color doppler ultrasonography, inguino-scrotal swelling, poor assessment / misdiagnosis, pediatrics, infarcted testicle, undescended testicle, testicular torsion

## Abstract

In this report, we discuss the case of a four-year-old boy known to have global developmental delay (GDD) and infantile spasm. The child was brought to the emergency department (ER) with a tender inguinal swelling and fever. Notably, there was no previous indication of an undescended testicle (UDT), and the scrotum was not examined in the ER initially. The abdominal radiograph was unremarkable, and an ultrasound of the groin was requested to investigate the nature of the swelling. The ultrasound yielded a differential diagnosis of direct or indirect inguinal hernia containing intestinal loops or a necrotic lymph node. Ultimately, scrotal examination and repeated imaging confirmed the diagnosis of bilateral UDT with torsion and suspected infarction on the right side.

Both UDT and testicular torsion (TT) are prevalent genitourinary disorders. However, the occurrence of torsion in an undescended testis is not commonly observed or documented, particularly within the age group presented in our case. TT is a surgical emergency, and its prognosis relies on early recognition and management in order to salvage the testis. In this case, there was a missed examination opportunity; therefore, it is imperative for emergency physicians to routinely include scrotal examination as part of the physical assessment when evaluating children with abdominal or groin pain in order to promptly identify such cases.

## Introduction

Undescended testis (UDT), or cryptorchidism, can be described as the failure of the testicle to descend to the scrotum. Testicles that have not descended into the scrotum can have long-term complications, including infertility [1]. The prevalence of UDT is quite common, as it has been found to affect between 2% and 8% of boys at birth [1]. Coincidentally, UDT has a strong association with hernias. Inguinal hernias were present in 90% of newborns with UDT [2].

In this study, we explore the atypical presentation of a four-year-old child with a tender inguinal swelling associated with fever and constipation. Initially, TT was not suspected. The ramifications of a misdiagnosed torsed UDT are significant; however, with early management, including clinical examination and ultrasound imaging, confirmation of the diagnosis may be vital in salvaging the testis.

## Case presentation

A four-year-old boy with a known history of global developmental delay (GDD) and infantile spasm was brought to the emergency department (ER) with fever and sudden onset inguinal swelling noticed by his mother. The fever was of one day duration, reaching 38°C, which responded to antipyretics. Additionally, it was associated with two episodes of vomiting, and there was a positive history of chronic constipation, with the last bowel movement being two days ago. On abdominal examination, a right inguinal swelling measuring 4x4 cm in diameter was noticed, which was warm, non-reducible, and tender. There was no note of any scrotal examination findings at this stage.

**Figure 1:**
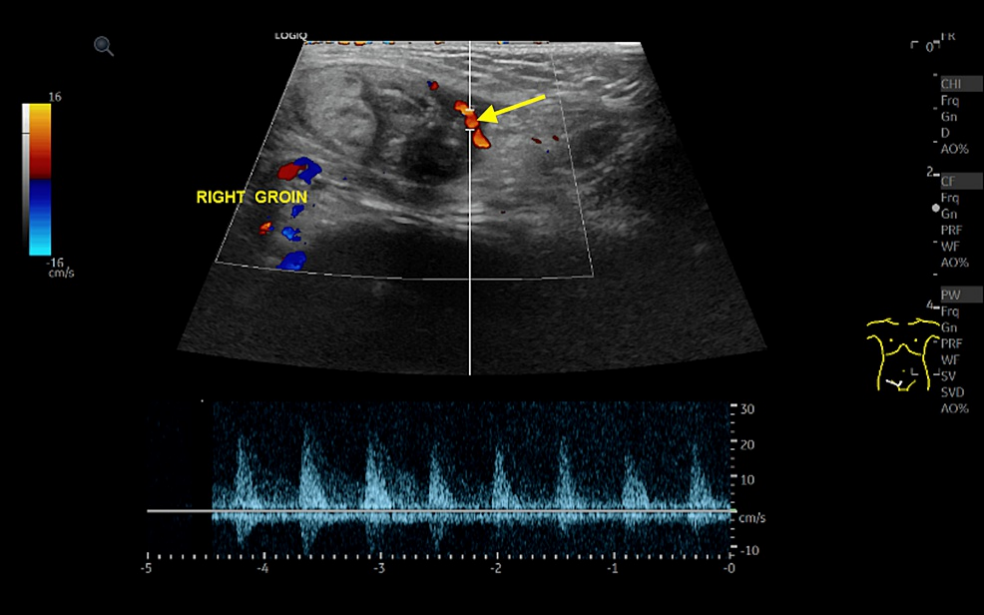
A doppler ultrasound image of the right groin shows an oval-shaped heterogeneous structure with peripheral vascularity (yellow arrow).

The on-call resident radiologist was called, and a gray scale and doppler ultrasound were done (Figure 1). The findings were of a subcutaneous heterogeneous lesion in the right groin, measuring about 3.2 x 1.6 cm, with suspicion of intra-lesional tubular-like structures and internal tiny echogenic shadowing focus. The question of possible communication to the inguinal region was raised. There was no surrounding fluid and no definite mobility of the inner structures in the provided images. Peripheral vascularity was noted. It was proposed at that time that the findings could represent a necrotic inguinal lymph node.

After assessing the patient, the pediatric surgical team recommended no surgical intervention and instead referred the patient to pediatric medicine for further management. A note is again made that, unfortunately there was no documentation of any scrotal exam being done.

On the next day, the senior staff radiologist reviewed the ultrasound images and suggested right inguinal hernia containing bowel loops as another possible differential diagnosis. The patient was called back for a re-scan. Meanwhile, the surgical team had re-examined the patient, this time including the scrotum, and had found an empty scrotum with non-palpable testes.

**Figure 2:**
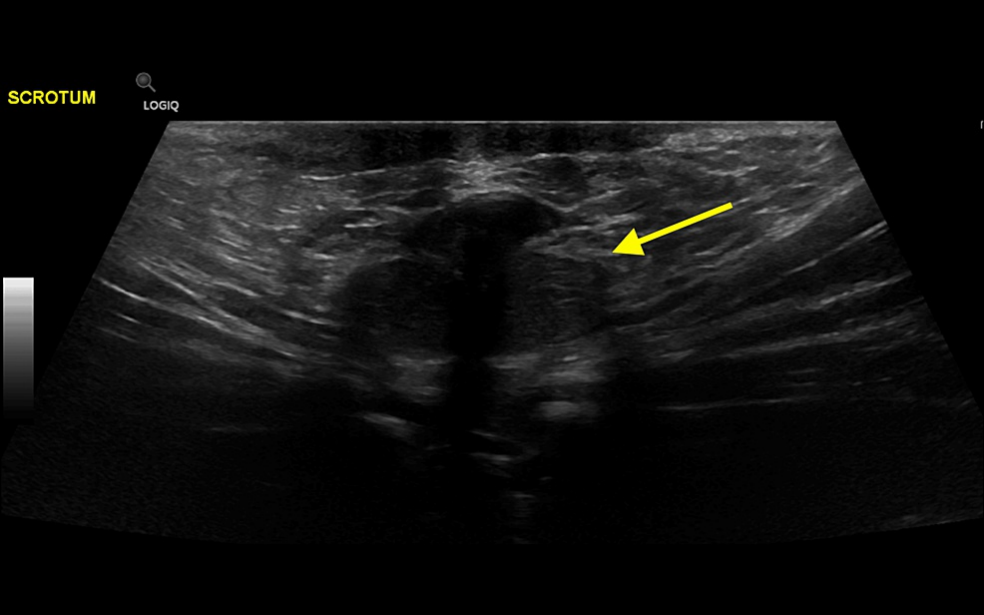
Ultrasound image of the scrotum shows empty scrotum (yellow arrow).

**Figure 3:**
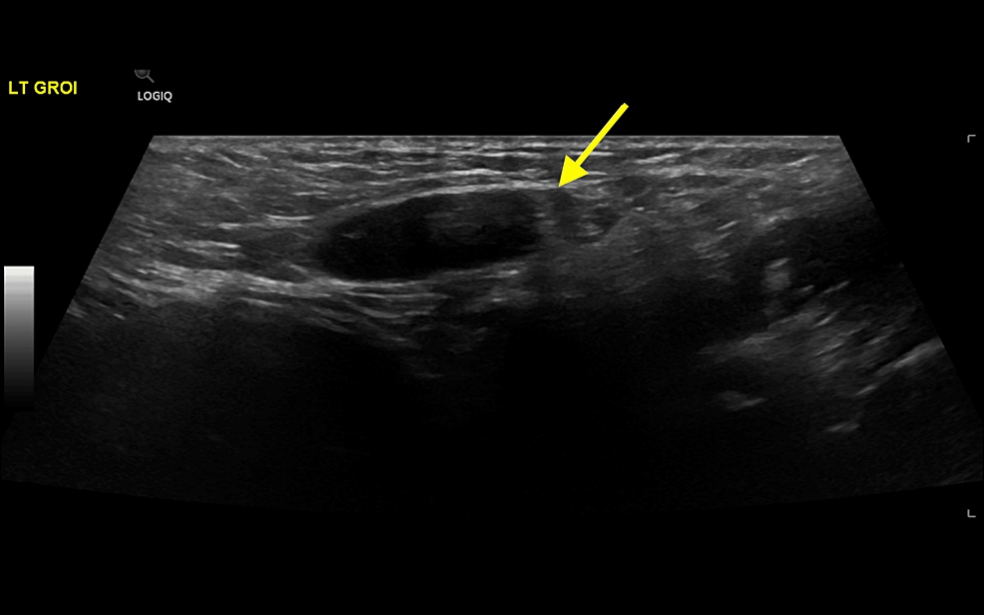
Ultrasound image of the left groin shows a small inguinal testicle (yellow arrow).

**Figure 4:**
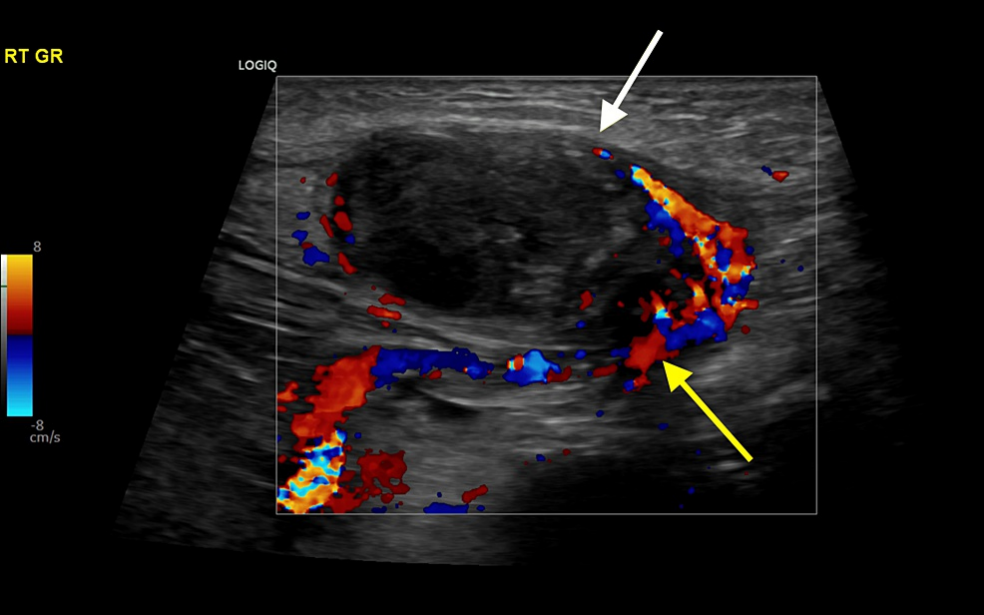
A color doppler ultrasound image of the right groin shows a heterogenous hypoechoic oval-shaped structure representing an infarcted testicle (white arrow), with peripheral vascularity representing a twisted spermatic cord (yellow arrow).

The repeated ultrasound involved a full scrotal and inguinal scan. It showed an empty scrotum (Figure 2). The left groin showed small but otherwise normal looking testicle (Figure 3), and the right showed the oval shaped heterogeneous structure with mixed internal heterogeneousity and absent internal flow on color doppler. Circumferentially, there was increased flow at the periphery with traceable flow from the lower pole, going toward the inguinal vessels, most likely in keeping with testicular torsion and even possibly infarcted inguinal testicle (Figure 4).

The case was urgently referred to the pediatric urology team and was booked for urgent examination under anesthesia (EUA). Surgical exploration revealed a gangrenous right testicle. The patient underwent EUA with a right inguinal simple orchidectomy and a left inguinal orchidopexy and meatotomy. 

The patient was well and stable postoperatively, with an uneventful recovery. He was discharged from the hospital on the second day and followed up after three weeks in the pediatric urology clinic with no concern. He is following up with the epilepsy clinic regularly and is doing well.

## Discussion

A warm and tender inguinal swelling in a child is a serious complaint that requires urgent evaluation to rule out the differentials. Among the differentials are incarcerated inguinal hernias and TT [3]. Initially, inguinal hernia was suspected due to the irreducible inguinal mass associated with episodes of vomiting and constipation. However, it was unlikely, as there was no history of abdominal distention and no suggestion of bowel obstruction or gas in the groin on the abdominal radiograph.

TT has a bimodal age distribution, with peak incidence in the first year of life and puberty at ages 10 to 14 [4]. The annual incidence of TT was found to be 3.8 per 100,000 patients under 18 years old [5]. Patients will present with severe scrotal pain associated with nausea and vomiting [6]. It is a urological emergency due to the twisting of the spermatic cord within the scrotum, limiting blood flow to the testicle. This classical presentation allows for prompt diagnosis and surgical intervention. 

Atypical presentations of TT may occur, with symptoms often being non-specific [7]. This is especially true in our case, as he was not able to communicate his symptoms due to longstanding GDD and had other misleading co-occurring symptoms, such as fever. A missed diagnosis or delay in the management of a torsed UDT can result in significant complications, namely testicular necrosis and infertility [8]. 

In this case, there were multiple factors that led to the delay in diagnosis, which eventually resulted in the need for orchidectomy, the most important being the lack of an initial scrotal examination. It is imperative to examine the external genitalia in patients presenting with inguinal swelling and pain [9], as it would have revealed an empty scrotum. As for imaging, it is also vital to include the scrotum when sonographically scanning an inguinal lump in a boy. It is widely recognized that color Doppler ultrasound is the best imaging modality for the initial evaluation of a scrotal pathology, especially if it is clinically unclear [10].

Doppler ultrasound (DUS) is a useful tool to assess the testicular blood flow if the diagnosis of TT cannot be confirmed clinically [11]. A study done in 2021 has shown that the use of DUS in patients suspected of TT has significantly reduced the frequency of negative surgical exploration without increasing the rate of missed testicular torsion [12].

The gold standard when diagnosing TT remains surgical exploration [13]. However, the prognosis and success rate of surgical intervention are heavily dependent on time. The reported success rate is 90% to 100% for surgeries done within six hours; however, it drops to 25% to 65% within 6 to 12 hours and 0% to 25% within 12 to 24 hours [14]. The ramifications of a delayed or missed diagnosis may result in physicians resorting to surgical exploration in order to rule out TT definitively [12].

## Conclusions

A torsion of an UDT in a child can be a challenge to diagnose. The ramifications of a missed diagnosis are significant; therefore, a clinical examination followed by imagining should be done urgently in order to prevent ischemia and infarction of the testicle.
